# Sustainability Evaluation of mHealth Apps and Wearables: Protocol for a Systematic Review

**DOI:** 10.2196/68649

**Published:** 2025-10-10

**Authors:** Sehrish Khan, Pantea Keikhosrokiani, Kaisa Kauppinen, Sharon Guardado Medina, Minna Isomursu

**Affiliations:** 1 Faculty of Information Technology and Electrical Engineering University of Oulu Oulu Finland; 2 Faculty of Medicine University of Oulu Oulu Finland

**Keywords:** sustainability, sustainable health care solutions, mHealth solutions, mobile health apps, wearables

## Abstract

**Background:**

Mobile health (mHealth) technology is widely used in health care systems globally to improve patients’ quality of life and reduce the burden on caregivers. Mobile apps allow users to manage different health-related situations from their homes, such as self-monitoring their symptoms, keeping track of progress, and maintaining a profile for a particular condition. However, the benefits of mHealth apps are compromised due to a lack of sustainability evaluation. Currently, there is a lack of all-inclusive research that presents sustainability evaluation from economic, social, and environmental perspectives of mHealth and wearables. Thus, it is important to evaluate the sustainability of newly proposed or already in-use mHealth solutions.

**Objective:**

The objective of this systematic literature review is to deeply understand and analyze different dimensions of sustainability in the health care domain and identify existing evaluation criteria and frameworks for mHealth solutions.

**Methods:**

This protocol was developed following the PRISMA-P (Preferred Reporting Items for Systematic Review and Meta-Analysis Protocols) guidelines. A planned and systematic approach will be used to identify, evaluate, and synthesize relevant studies reported in English. We have conducted a systematic search focusing on search terms related to sustainable health care, mHealth solutions, and evaluation or assessment criteria in digital databases of Scopus, Web of Science, ACM, PubMed, PubMed Central, and CINAHL.

**Results:**

As of September 19, 2024, preliminary searches have been conducted, identifying 1368 relevant papers that meet the inclusion and exclusion criteria and are eligible for further screening.

**Conclusions:**

This protocol will guide the review of existing literature on essential aspects of the sustainability of mHealth solutions and evaluation frameworks. To the best of our knowledge, the proposed systematic review will be the first review to extensively study essential concepts related to defining sustainable mHealth solutions, identifying key dimensions founding the basis for evaluation frameworks, and discussing the existing sustainability evaluation frameworks for mHealth solutions.

**Trial Registration:**

PROSPERO CRD42024599333; https://www.crd.york.ac.uk/PROSPERO/view/CRD42024599333

**International Registered Report Identifier (IRRID):**

DERR1-10.2196/68649

## Introduction

### Background

The World Health Organization (WHO) defines mobile health (mHealth) as “medical and public health practice supported by mobile devices, such as mobile phones, patient monitoring devices, personal digital assistants (PDAs), and other wireless devices” [1]. mHealth innovations can provide quality health care services to the public and health care professionals [2]. These applications serve multiple purposes, such as monitoring, management, and communicating health-related information between a patient and health care provider [3]. Additionally, mHealth technology promises to reduce the burden of meeting in-person health care needs of a growing population, especially older people, by providing facilities like remote consultation and online appointments in accordance with the needs of patients [4]. Theoretically, mHealth solutions are considered a key to strengthening the health care system.

### The Concept of Sustainability in the Health Care Domain

Sustainability is defined in terms of creating viable ecological conditions for humans and nature on this earth so that our existence can be ensured for an indefinite time [5,6]. The Brundtland Report, World Commission on Environment and Development (1987), defines sustainability as “meeting the needs of the present without compromising the ability of future generations to meet their own needs” [7]. In terms of mHealth in the health care sector, sustainability is further assessed through economic, social, and environmental dimensions [8,9]. Therefore, the sustainability of mHealth is defined in the literature as follows:

“*Sustainability rests on three components: social, economic, and environmental. In the mHealth sector, this translates into assessing the social, economic, and environmental effects of mHealth innovations*” [9].

Social aspects ensure that mHealth solutions are accessible, equitable, and culturally appropriate for diverse populations. Economic sustainability mainly focuses on the cost-effectiveness, affordability, and long-term financial viability of mHealth interventions. The environmental aspect of sustainability considers the ecological impact of mobile technologies, including energy use, electronic waste, and sustainable device lifecycles.

### Lack of Sustainability Evaluation for mHealth Solutions

In 2019, around 2.5 billion people used smartphones worldwide, and 50% had a mHealth app installed [10]. Unfortunately, many users skip using mHealth apps after a short period [9]. One of the reasons behind this behavior is a lack of sustainability evaluation of mHealth solutions during the development and deployment phase, and the absence of a plan for sustainable follow-up action [2]. Moreover, the short lifespan of mHealth solutions may be due to financial structures with misaligned incentives and rapid technological advancements.

The concept of sustainable health care is gaining widespread attention from scholars and health care professionals. In the past, sustainability was associated with environmental aspects such as reducing carbon footprints and waste from product development and processes for health care facilities [11]. However, the latest research findings suggest that sustainability in health care needs to be explored from economic, social, and environmental aspects [9].

Literature [9,12-16] also highlights the importance of evaluating the sustainability of mHealth solutions and a lack of sustainability evaluation frameworks. Moreover, a number of mHealth solutions fail in the implementation and follow-up stage due to ineffective guidelines and regulations for sustainable design [2]. Another study points out that the need for sustainability from the economic and financial perspective limits the long-term and regular use of mHealth solutions by patients [17].

To date, no literature review has provided a comprehensive study on sustainability evaluation frameworks for mHealth apps and wearables together, with a focus on economic, social, and environmental aspects in health care settings.

Toward the goal of successfully studying, identifying, and analyzing these existing studies, we aim to systematically review the concept of sustainability in the health care domain and sustainable mHealth apps and wearables and discuss the success factors in developing an evaluation framework for sustainability. The theoretical assumption of this study is that a sustainability evaluation framework involving 3 pillars of sustainability (economic, social, and health environmental) will assist all stakeholders in achieving the goals of sustainable mHealth solutions.

### Research Questions

In this paper, we study the concept of sustainability in health care for mHealth apps and wearables. This review aims to assess the sustainability and benefits of mHealth in terms of economic, social, and environmental sustainability. We investigate key determinants of sustainability from economic, social, and environmental factors for mHealth solutions.

To address these aims, we have set the following research questions:

1. How is sustainability defined for mHealth solutions in the context of digital health care?
2. What are the dimensions and elements of evaluating mHealth solutions’ sustainability from economic, social, and environmental perspectives?
3. What metrics, tools, frameworks, and models have been proposed to evaluate the sustainability of mHealth solutions, and what methods have the studies used to propose them?

### Objectives

To answer our research questions, we have identified the following objectives for the study. First, we will perform a thorough study of existing literature that defines sustainability in the health care domain, particularly for mHealth solutions, which include mHealth apps and wearables. Second, we will identify different dimensions and aspects related to evaluating sustainability, which form the basis for designing sustainability evaluation frameworks. Lastly, our objective is to investigate and identify the sustainability evaluation frameworks and models for mHealth solutions reported in the literature.

## Methods

### Ethical Considerations

In this protocol paper, we are not required to acquire ethics board approval as defined in the guidelines from the Ethics Committee of Human Sciences [18]. We have followed the University of Oulu ethics process for writing and submitting the protocol paper.

### Study Design

**Figure 1:**
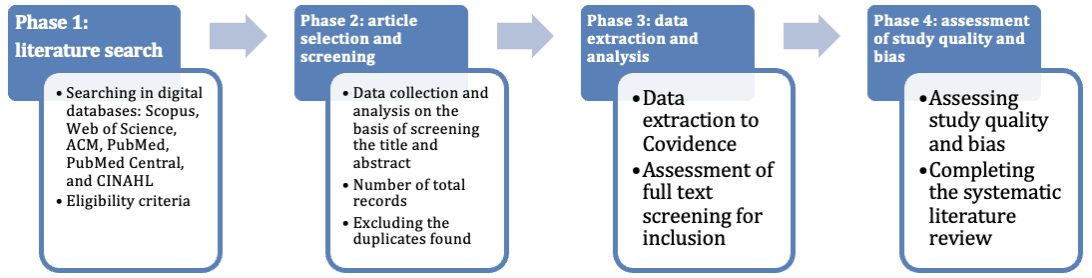
Illustration of the study structure.

This protocol is reported according to the PRISMA-P (Preferred Reporting Items for Systematic Review and Meta-Analysis Protocols) 2015 guidelines [19]. The protocol is registered on the International Prospective Register of Systematic Reviews (PROSPERO CRD42024599333). PRISMA-P serves as a guideline for researchers to fulfill a checklist for preparing and documenting protocols for systematic literature reviews. The study structure is provided in Figure 1 and explained in detail in the next sections.

### Study Participants

In this systematic review, we will include all studies that describe mHealth solutions in the form of mHealth apps and wearables for individuals or groups to improve health care quality and access to health care services. Study participants will represent all ages, genders, ethnicities, employment statuses, occupations, and roles in the reviewed study (ie, patients, health care professionals, informal caregivers, researchers, designers, and policymakers in health care).

### Literature Search

A systematic search for papers published between 2012 and September 19, 2024, was performed in the digital databases of Scopus, Web of Science, ACM, PubMed, PubMed Central, CINAHL, and Google Scholar. During our initial search in these databases, it was observed that most of the research papers relevant to the scope of this study were published from 2012 onward up until 2024. Thus, we selected the time filter to be from 2012 and 2024. The keywords and terms used for searching in these databases are presented in Table 1.

**Table 1:** Search terms used for data collection.

Field of interest	Search string
Sustainable health (keywords related to sustainability)	(“sustainability” OR “sustainable” OR “sustainable health” OR “social sustainability” OR “economic sustainability” OR “environmental sustainability” OR “ecological sustainability”)
mHealth apps and wearables	(“mHealth“ OR ”m-Health“ OR ”mobile health“ OR ”mobile healthcare“ OR ”wearable device“ OR ”wearable technology“ OR ”wearable sensor“ OR ”smart wearable“ OR ”medical wearable“ OR ”healthcare wearable“ OR ”remote monitoring device“ OR ”biosensor“ OR ”fitness tracker“ OR ”wearable telehealth“
Evaluation methods	(“design” OR “development” OR “Framework” OR “model” OR “assessment” OR “evaluation”)

The search strings focus on three main areas suitable for the systematic literature review discussed in this protocol paper: (1) keywords related to sustainable health, including the 3 sustainability pillars; (2) keywords related to mHealth solutions, which include mHealth apps and wearable devices; and (3) keywords related to evaluation frameworks existing in the literature. An initial search in the digital databases resulted in 1368 papers in total.

### Eligibility Criteria

Eligibility will be described by defining the criteria to include and exclude research papers in the literature review. Studies will be selected according to the criteria described in Textbox 1.

**Eligibility criteria.**

Inclusion criteria

- Peer-reviewed articles, conference proceedings, and book chapters.
- Papers available in full text.
- Papers that describe only quantitative, qualitative, or mixed research methods actually evaluating the sustainability of mHealth apps.
- Papers that define [9] and discuss the concept of sustainable health care for mHealth apps and wearables. Examples of mHealth studies without wearable devices include [11,20], which assess mHealth app sustainability, and [21], which evaluates an mHealth app with a wearable.
- Papers focused on economic, social, and environmental sustainability perspectives in the health care domain for mHealth apps and wearables.
- Papers that present or propose an evaluation framework or assessment method for evaluating the sustainability aspects of mHealth and wearables.

Exclusion criteria

- Papers that are purely study protocols, systematic literature reviews, or scoping reviews; abstracts; posters; and short papers.
- Papers that are not in English.
- Papers that are not research articles or publications or are otherwise irrelevant to the topic.
- Papers that are not related to mHealth solutions.
- Papers that only focus on the theoretical definition of sustainable health care rather than providing or doing a sustainability assessment for mHealth solutions.
- Studies on nonsustainability-related evaluation of mHealth apps.
- Studies on the usability or exploration of mHealth apps and wearables that do not address the topic of sustainability evaluation.

### Article Selection and Screening

The initial search results from different databases were imported into Covidence, a web-based tool for analyzing systematic reviews [22]. Covidence allows importing the results from various databases and removes the duplicates. Later, the papers included will be first screened by looking at the title and abstracts, keeping the eligibility criteria in mind. The abstracts of all the included papers will be screened by 4 authors (SK, PK, KK, and SGM). Then, a second round of full-text screening will be performed keeping the eligibility criteria in perspective by the same authors. Any disagreements regarding the inclusion or exclusion of studies will be resolved through discussion. The disagreements will be discussed and resolved with mutual consent.

### Data Extraction and Analysis

Key information from the articles included will be extracted using the data extraction form provided in Covidence. Prior to data extraction, SK will perform a pilot on a sample of 5 included papers to adjust any changes. Data extraction will be performed independently by 2 reviewers (SK and KK or SK and SGM). Any disagreements will be resolved by discussion.

### Assessment of Study Quality and Bias

Quality assessment will be produced in parallel to the data extraction process using a quality assessment form included in Covidence. The study bias assessment will be performed using the checklist proposed by Dybå and Dingsøyr [23] and Yang et al [24]. For each included study, we will report our assessment of the risk of bias (ie, low, high, or unclear risk for each domain) and provide a concise description explaining the reason behind our judgment. Any discrepancies regarding the judgment of the bias of a study will be resolved through mutual discussion among authors.

### Data Analysis and Synthesis

This systematic review will study different dimensions of sustainability evaluation and discuss existing frameworks for sustainability evaluation of mHealth solutions. In the included studies, the frameworks for sustainability evaluation will be addressed, and the methods used in the studies to extract the framework will also be discussed. The included publications can be based on quantitative, qualitative, or experimental data. Thus, data synthesis will be performed using quantitative and qualitative data from the full texts of the papers included. To perform data synthesis, a thematic approach will be used to identify and create categories from the results based on similarities and patterns found in different themes. These themes will help extract information about definitions and evaluate criteria or existing frameworks for sustainability in mHealth solutions. Overall, the entire search strategy from search and article selection to data extraction and data synthesis will be piloted using 5 sample papers chosen randomly from the included papers.

## Results

As of September 19, 2024, a total of 1368 papers have been found to meet the inclusion criteria defined in Textbox 1. These papers have been imported into Covidence for further screening and analysis.

## Discussion

### Anticipated Findings

This systematic review focuses on addressing important concepts related to the sustainability of mHealth solutions. In the health care setting, mHealth solutions claim to strengthen the health care system and its capacity without compromising on the quality of care given to patients. Currently, the growing population presents many challenges to health care systems globally in terms of reducing costs and allocating resources optimally while ensuring high quality of provided services. In such a scenario, caregivers feel a heavy burden of giving quality treatment to patients. mHealth solutions can help in many ways, such as through facilities like remote consultation, online video appointments, maintaining profiles of patients in portfolios, and engaging patients in their personalized treatment journey. However, in the absence of sustainability evaluation of mHealth solutions, these benefits will be undermined.

Hence, this literature review aims to report different concepts related to sustainable health care related to mHealth solutions, such as definitions, aspects, and sustainability evaluation criteria or frameworks. Also, we aim to study the significance of economic, social, and environmental perspectives of sustainability evaluation. Currently, there is a broad scope for exploring the concept of sustainable health care from economic, social, and environmental perspectives. Thus, the systematic analysis of existing evaluation frameworks will help in the future to create evaluation frameworks specifically for the sustainability evaluation of mHealth solutions. This protocol’s primary contribution lies in defining the research gap and the research questions, as well as in its structured approach proposed for the literature review to address the gap. This gap focuses explicitly on assessing the sustainability of wearables and mHealth apps and providing guidelines for reviewing existing literature on the subject.

### Limitations of This Study

First, this protocol is limited to studies published in English and subject to full text availability. Second, following standardized procedures to include and exclude studies and extract and analyze data, some degree of individual judgment is unavoidable, as is the case in other systematic literature reviews. Third, while there are some definitions of sustainability in the context of mHealth apps, the lack of a universally accepted definition can result in differences in the design of the included studies and the way they have measured the outcomes and reported the results. Lastly, this study is limited to the papers published from 2012 to 2024.

## Abbreviations

mHealth: mobile health
PRISMA-P: Preferred Reporting Items for Systematic Review and Meta-Analysis Protocols
PDA: personal digital assistant
WHO: World Health Organization
